# Cone-beam computed tomography of the head in standing equids

**DOI:** 10.1186/s12917-019-2045-z

**Published:** 2019-08-13

**Authors:** M. D. Klopfenstein Bregger, C. Koch, R. Zimmermann, D. Sangiorgio, D. Schweizer-Gorgas

**Affiliations:** 1 Swiss Institute of Equine Medicine (ISME), Department of Clinical Veterinary Medicine, Vetsuisse-Faculty University of Bern, and Agroscope, Bern, Switzerland; 2 Division of Clinical Radiology, Vetsuisse-Faculty, University of Bern, Bern, Switzerland

**Keywords:** Cone beam computed tomography, O-arm®, Horse, Standing, Head

## Abstract

**Background:**

Computed tomography in standing horses has revolutionized diagnostic imaging. The O-arm®, a cone beam computed tomography (CBCT) scanner with a gantry opening of 96.5 cm is routinely used for image-guided spine and neurosurgery in humans. The aim of this study is to describe the set-up and first experiences using the O-arm® to achieve CBCT imaging of the head in standing horses.

CT imaging of the predefined region of interest (ROI) was tested on 2 cadaveric heads, concentrating on centering issues within the gantry, as well as determining the number of scans needed per ROI. All horses presented with head-related diseases and subjected to a CBCT examination between February 2015 and November 2016 for CBCT were included. Per scan, a limited field of view, i.e. a cylindrical volume of 21 cm in diameter and 16 cm in height was acquired within 13 s. Depending on the dimensions of the ROI, the minimum number of scans could range from one to six, if the entire equine head is to be examined in an adult horse.

**Results:**

Sixty-eight horses were included, five of which had a follow-up CBCT exam, and two of which were presented twice for two different indications (75 clinical cases). A total number of 449 acquired three-dimensional (3D) scans were recorded for these 75 cases. Two-hundred and forty-two 3D scans (54%) were considered as diagnostic quality. The imaging procedure was generally well tolerated by the sedated, standing equid, and diagnostic studies were performed in 73 out of 75 cases (97.3%). Motion artefacts and inadequate centering of the ROI were the most common reasons for non-diagnostic quality images and repeat scans of the same ROI.

**Conclusions:**

CBCT is a valuable imaging modality for the equine head. Advantages of the O-arm® compared to a conventional multi-slice helical CT for imaging of the head in standing equids include the rapid image acquisition, the gantry’s mobility in all dimensions, and the free movability of the entire imaging unit. Disadvantages include the considerable sensitivity to motion artefact, increased scatter, low soft tissue contrast and the limited dimensions of the field of view.

## Background

Computed tomography (CT) has become an important imaging modality for the diagnosis of diverse head disorders in the horse [1–6]. The cross-sectional imaging modality provides images of nasal and paranasal passages, the teeth, skull bones, the hyoid apparatus, and the teeth without superimposition and allows 3-dimensional reconstruction [7]. Overcoming the restrictions of two-dimensional radiographic imaging, CT imaging has become the gold standard imaging modality to diagnose common disorders in the equine head and to perform pre-surgical planning. To avoid general anesthesia, CT of the head is increasingly performed with the horse in a standing position [1–4], [8–11].

While different technical set-up’s using conventional helical CT units in the standing horse have been described, two distinct techniques in principle are established; either with a sliding gantry that passes over the fixed head of the equid, or with a stationary gantry through which the head is moved at a constant speed by placing the equid on a platform that is suspended by air castors [9]. More recently, CT scanners using cone beam technology have been introduced to the equine market (Pegaso™, Epica Medical Innovations, San Clemente, USA; Equimagine™, Equine 4DDI, Universal Medical Systems, Solon, USA). The cone-shaped x-ray beam in a cone-beam-CT (CBCT) uses a large-area detector plate obtaining fully volumetric data from multiple projections. All projections are acquired in a single rotation around the patient without moving the patient through the scanner [12].

The CBCT scanner used in the present study is designed and FDA-approved for use in a surgical environment (O-arm®, Medtronic Inc.). It is a transportable scanner, which does not require a fixed installation or a separate, specific power supply. The gantry diameter is 96.5 cm wide and can be opened to a window of 69.9 cm at the telescoping door. The gantry is mobile in all three dimensions and can be tilted around its horizontal and vertical axis [13].

The imaging mode of the O-arm® is either in a single plane, producing 2D pulsed fluoroscopic images at a rate of 30 frames/second, or generating volumetric data with 192 images acquired during a 360 ° rotation within 13 s. Image reconstruction of the 30 × 40 cm activated Si/CsI digital flat panel detector results in a cylindrical volume of 21 cm in diameter and 16 cm in height with an acquisition matrix of 512 × 512, and a resolution of 0.415 × 0.415 × 0.833 mm; pixel pitch of 0.194 mm.

In humans, CBCT is routinely used for imaging dental and bone structures of the maxillofacial region [14], whereas in veterinary medicine, information is limited. To date, available literature has concentrated on the use of CBCT for dental abnormalities in dogs, cats and rabbits [15–18].

Mobile CBCT units, such as the O-arm®, with a large-bore, highly maneuverable gantry and rapid image acquisition without gantry-movement in relation to the subject, undoubtedly provide advantages of particular importance for diagnostic imaging of sedated, standing equids. However, there are also conceivable disadvantages inherent to the cone beam geometry such as increased scatter radiation reducing the contrast resolution, the fixed field of view, and that subject-motion during image acquisition affects the whole volume acquired. As yet, the use of CBCT for imaging standing, sedated equids has not been critically assessed.

The aim of the present study was to evaluate if the O-arm® can be used reliably as an imaging modality for the diagnosis of head disorders in standing horses.

For this purpose, we first established the technical set up and examination protocol in cadaver heads and clinically normal horses. Subsequently, the established examination protocol was used for routine diagnostic CBCT imaging in a clinical setting. Here, we present a detailed description of the CBCT examination protocol, the technical installations needed, and our experiences with this imaging modality for the routine examination of head disorders in standing, sedated equids. Special attention is given to the number of scans acquired to provide images of diagnostic quality for the particular ROI, to allow for a radiological diagnosis to be established, or to exclude any structural changes relevant to the suspected underlying condition.

## Results

### Cadaver heads

The number of required scans per region of interest resulted in 1 to 6 scans.

To image a complete head, 6 cylindrical volumes were necessary (Table 1).

**Table 1:** Number of required scans per region of interest

Region of interest		Number of cylindrical volumes needed to image the region of interest
Premaxilla and pars incisiva	Maxillary and mandibular incisors and canini	1
Cheek teeth	Maxillary cheek teeth	2
	Mandibular cheek teeth	2
	Maxillary and mandibular cheek teeth	3–4
Sinus system	Nasal and paranasal passages incl. Maxillary cheek teeth	3
Mandible incl. The temporomandibular joint	unilateral	4
	bilateral	5
Temporomandibular joint	unilateral	1
	bilateral	2
Middle and inner ear, proximal hypoid apparatus	unilateral	1
	bilateral	2
Complete head		6

### Clinically sound horses

In live equids, the number of scans required per ROI (defined in cadaver heads), was not affected by variations in head position on the carbon table or within the gantry.

### Clinical cases

In total, 68 equids were subjected to a CBCT examination in the standing position during the study period. A follow-up CBCT examination was performed in five (7.4%) of the 68 equids, and a second CBCT examination of the head was performed in two equids for another distinct indication. In total 75 CBCT studies were included and reviewed.

The group of 68 equids examined included 34 mares, 30 geldings and four stallions with a mean age of 13.3 years (range 1 to 26 years). Forty-six were Warmblood horses, four Franches-Montagnes, three Ponies, two Standardbreds, two Lusitanos, and one each of the following: Thoroughbred, Arabian horse, Paint horse, Quarter Horse, Friesian horse, Tinker, Noriker, Haflinger, Icelandic Horse, Mérens and 1 donkey.

Equids were presented with the following clinical complaints and/or indications: uni- or bilateral nasal discharge (40), quidding/masticatory problems [9], head shaking [7], dental pathology identified during oral exam [4], trauma or congenital skull deformation [4], facial swelling [4], fistulation [3], cranial nerve deficit [2] and ataxia [2].

A total number of 449 acquired 3D scans were recorded for the 75 CBCT studies. The mean number of acquired CBCT scans per case was 6 (range 1–15 scans).

**Fig. 1:**
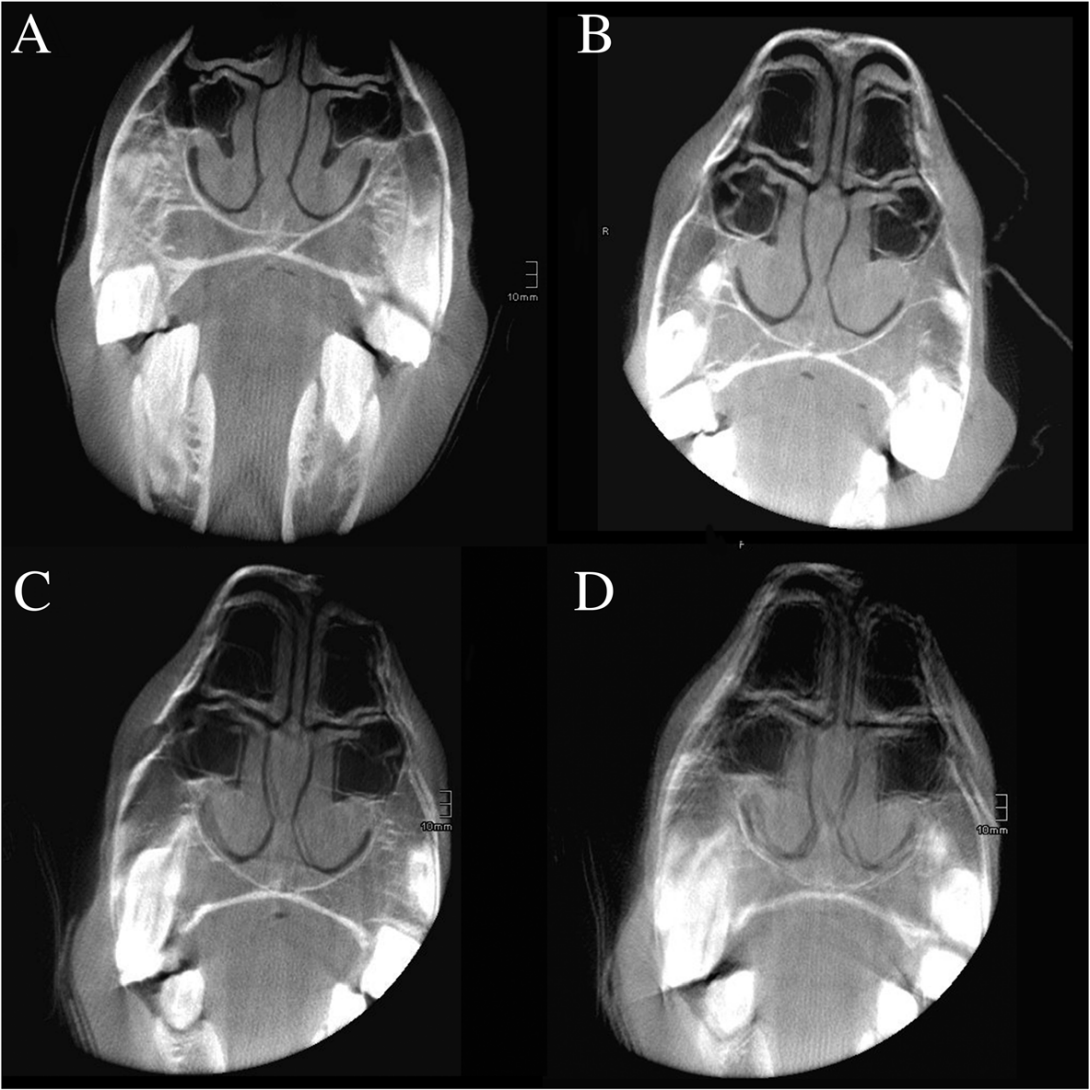
Illustration of the different categories of motion artefact. Transverse CBCT images of the nasal passages and nasal conchae illustrating the four different possible categories of motion artefact: **a** no or minimal motion artefact (category I), **b** mild motion artefact (category II), **c** moderate motion artefact (category III), and **d** marked motion artefact preventing diagnostic evaluation (category IV)

From these 449 scans, 242 (54%) were of diagnostic quality (cat. I-III). Fifty-nine (24%; cat. I) scans showed no or minimal motion artefact, 102 (42%; cat. II) mild motion artefact, and 81 (33%; cat. III) were of diagnostic quality despite moderate motion artefact (Fig. 1 a-c). Two-hundred and seven (46%; cat. IV) scans were declared as unfit for diagnostic purposes by a certified radiology technician and therefore not subjected to a radiologic evaluation (Fig. **1** d). In 2/75 (2.7%) CBCT examinations, all acquired scans were not of diagnostic quality (cat. IV), whereas in 73/75 (97.3%) of the cases CBCT scans of diagnostic quality were obtained.

**Fig. 2:**
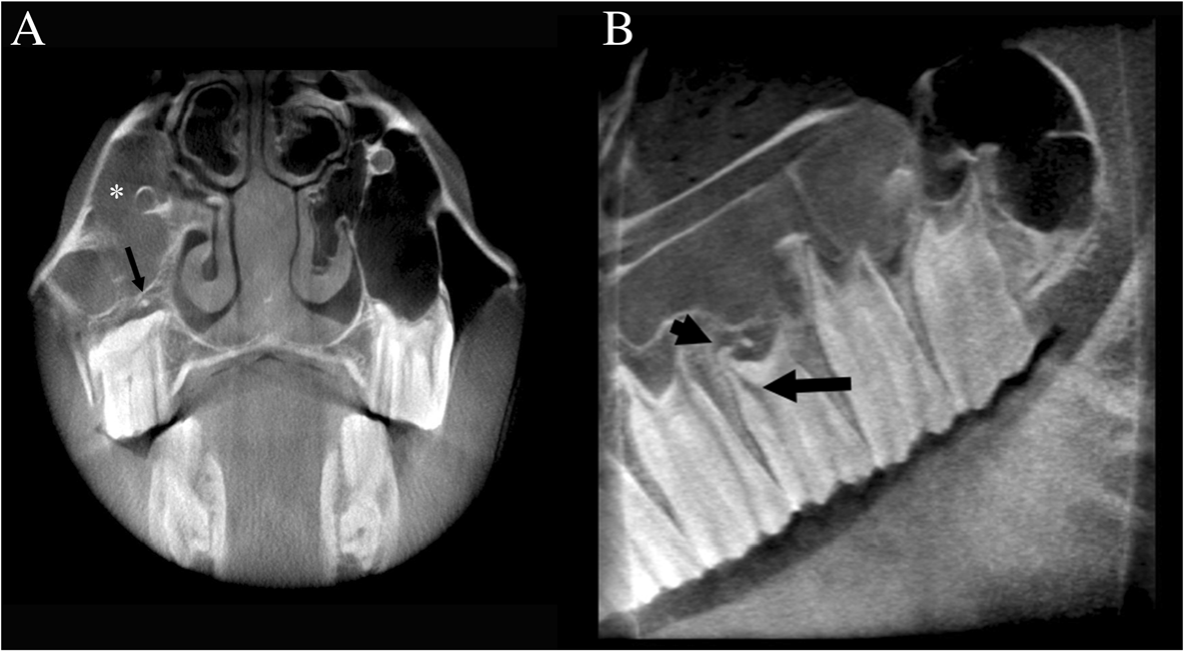
Thirteen-year-old Warmblood gelding with an apical infection of the tooth 109. **a** Transverse CBCT image at the level of 109; note the soft tissue attenuating material (asterisk) filling the rostral maxillary sinus and the presence of a small cementoma in the alveolar space at the level of the palatine tooth root (long black arrow). **b** Sagittal CBCT image through the right maxillary arcade: note the widening of the rostral pulp canal and the clubbing of the palatinal tooth root

**Fig. 3:**
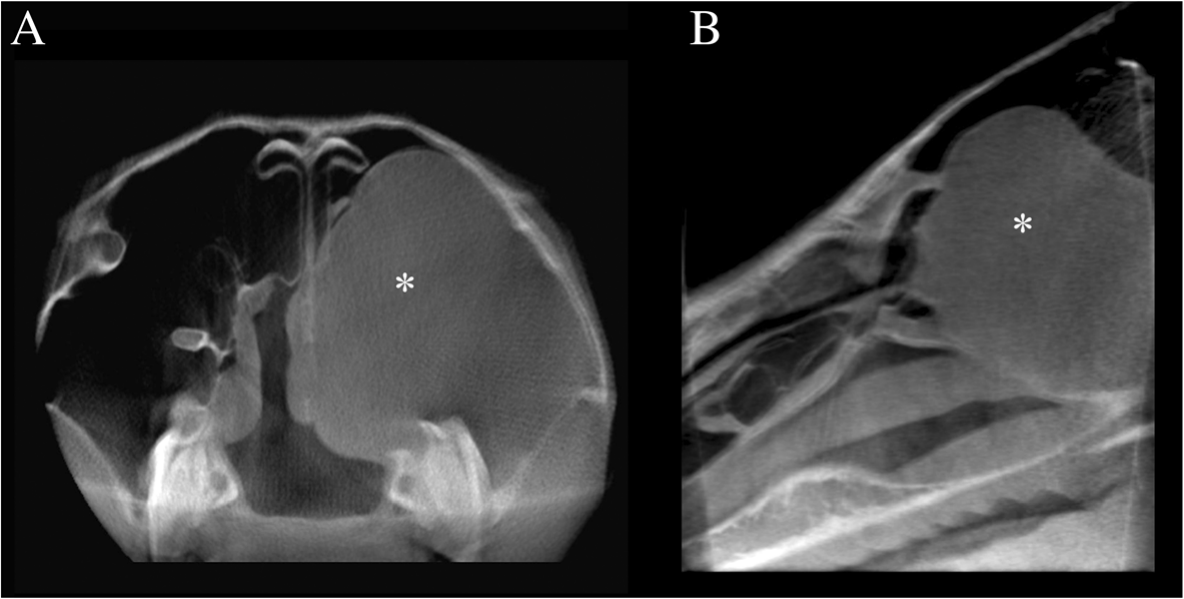
Thirteen-year-old Warmblood gelding with a space occupying lesion within the nasal and paranasal passages. Transverse (**a**) and sagittal (**b**) reconstruction: a well-delineated soft tissue attenuating space occupying lesion (asterisk) is visible in the caudal maxillary sinus, the conchofrontal sinus, and the nasal passages

**Fig. 4:**
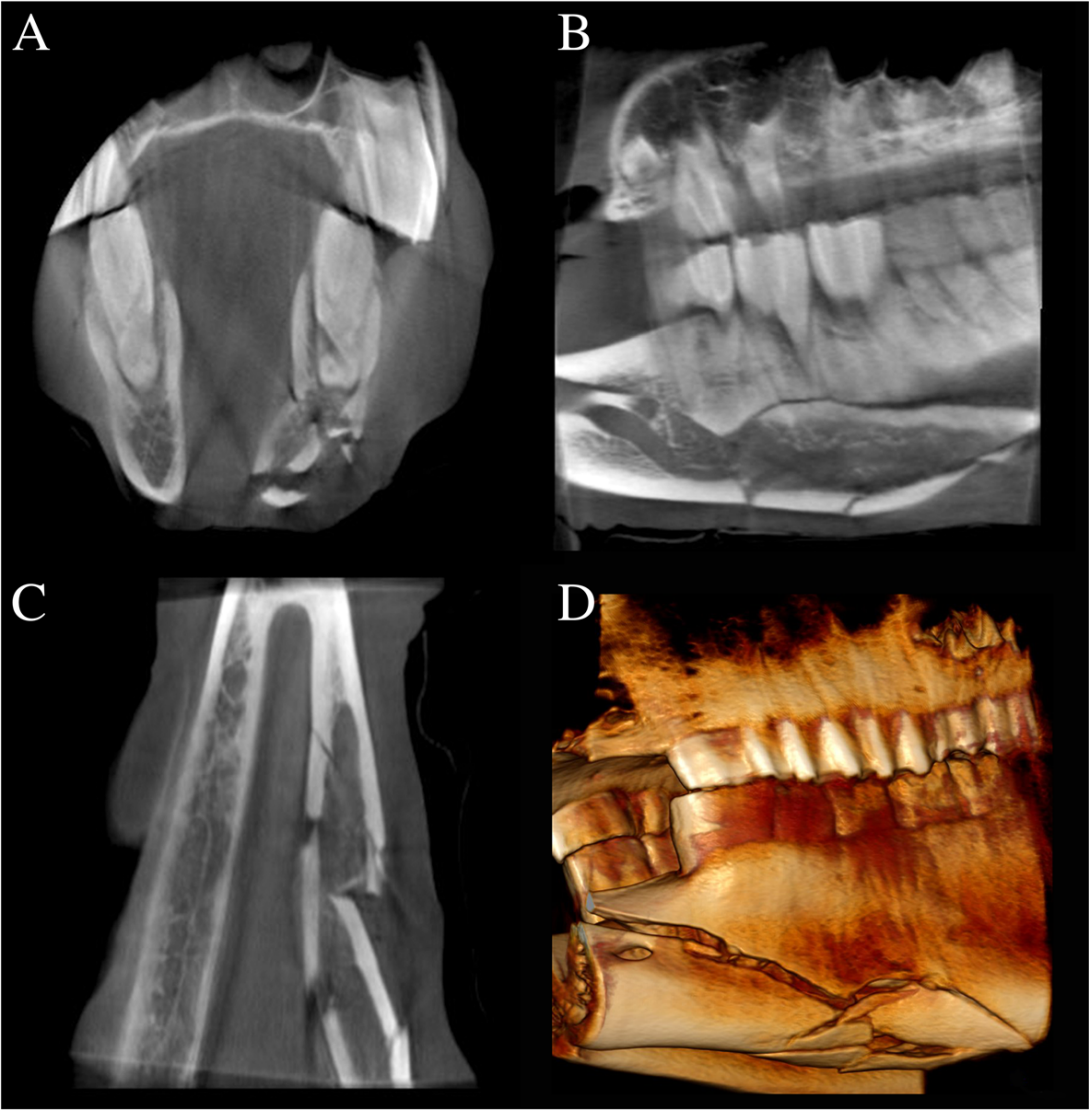
CBCT scan of a 20-year-old Akhal-Teke/Lusitano mix with a left comminuted mandibular fracture. **a-d** Transverse, sagittal and dorsal reconstruction and 3D volume rendering showing a left comminuted mandibular fracture in the diastema

The radiological diagnoses included primary dental disease (39) (Fig. **2**), with or without secondary sinusitis, space-occupying lesions in the nasal passages and paranasal sinuses [10] (Fig. **3**), fractures [7] (Fig. **4**), primary sinusitis [3], temporohyoid-osteoarthropathy [3], aggressive (lytic) bone lesions [2], suture periostitis [2], primary rhinitis [1], and soft tissue abscessation [1]. In an additional five cases, no structural changes were identified on scans of adequate diagnostic image quality, rendering a presumptive diagnosis of idiopathic headshaking in three horses. In one of the two remaining equids, a working diagnosis of soft tissue trauma with head tilt and ataxia was reached after ruling out structural bony abnormalities on CBCT images of adequate quality. No signs of inner or middle ear disease, nor masses in the central nervous system were seen in that case on the CBCT images. In the other remaining case, which presented chronic weight loss and a suspected abnormal feed intake, no abnormality was identified on CBCT imaging, and a megaoesophagus was subsequently diagnosed by means of contrast radiography.

### Image quality and artefacts

The CBCT imaging provides a high spatial and contrast resolution of bony structures, but a limited contrast resolution for soft tissues, when compared with conventional, helical multi-slicer CTs. The CBCT unit used in the present study did not allow diagnostic differentiation of different soft tissue qualities/densities.

## Discussion

This is the first detailed report on the use of a commercially available CBCT unit for the examination of the head in standing, sedated equids. We provide information about the CBCT unit and image acquisition, as well as our experiences from 75 clinical cases. Using the described set up and protocol, we were reliably able to obtain CBCT volume acquisitions of diagnostic quality that allowed the establishment of a radiologic diagnosis or to rule out structural changes in 73 of 75 cases (97.3%). The percentage of horses, in which no diagnosis could be established is comparable to the number reported using a conventional CT scanner with a sliding gantry, where no images of diagnostic quality could be acquired in 11 out of 114 horses [11]. However, the spectrum of established radiological diagnoses using CBCT seems to be larger enabling the diagnosis of temporohyoid-osteoarthropathy, aggressive bone lesions, suture periostitis, primary rhinitis and soft tissue abcessation beside the usual diagnoses like alveolitis with or without secondary sinusitis and fractures [11]. In this study, the accuracy of the established diagnosis was not assessed, but we still consider the use of CBCT as a valuable imaging modality to assess equine head disorders in standing sedated equids.

Positioning of the head in the gantry and restraint of the horse were comparable to conventional helical CT scanners [9, 19, 20]. Compliance with the examination procedure was good in the vast majority of equids. All horses in this study were sedated with detomidine only. This is contrasting wit other studies where a combination with opioids (specifically butorphanol) in addition to acepromazine half an hour before examination were used [9, 20]. The choice of using detomidine alone was based on clinical experience and advice of another clinical setting using the same protocol [21]. In our experience the effect of opioids may increase the likelihood of excitatory twitching head motions and an increased forward thrust in some equids for surgical procedures of the head, which could increase motion and hamper positioning within the gantry.

The acoustic noise generated by the rotating tube and flat panel detector was reduced by the use of earplugs. Scans were acquired in a minority of horses without earplugs as a few horses did not tolerate them. In these cases, background music or voices helped to distract the equid. Proper sedation and positioning of the horses allowed scanning of all horses without any personnel within the room, therefore exposure to radiation was negligible. It is worth mentioning that the dose level of a CBCT with the O-arm® is reported at around one-third of the dose of a conventional helical CT scanner [22].

The major challenge when using CBCT in standing sedated equids is to prevent motion of the examined equid during the 13 s of image acquisition. Even motion caused by breathing causes mild motion artefacts. In contrast to conventional CT, movement of the head causes motion artefact on all images reconstructed from the acquired volume, even if motion occurred only on a short moment of acquisition. Motion artefact was by far the most common reason to repeat scans and generate studies with multiple scans in equids. However, poor compliance hampered the acquisition of scans of diagnostic quality in only two horses. In all other horses, adequate sedation i.e. not too deep or superficial and a fixed position of the head within the vacuum cushion with additional taping of the head to the adjustable carbon table [21] decreased motion artefact in the other equids.

Another important limitation of the CBCT compared to conventional CT is the fixed cylindrical volume of 21 cm in diameter and 16 cm in length. Accurate positioning of the head and centering are therefore very important to image the planed ROI according to the clinical complaint. If the ROI exceeds the cylindrical volume, for example in horses affected with head shaking, at least six scans have to be acquired to cover the complete head to exclude structural changes. Currently, datasets from different scans cannot be linked. Therefore, during evaluation, one has to switch between different volume acquisitions to appropriately assess all areas of interest.

The cone beam architecture of the X-ray beam causes a higher amount of scatter radiation compared to conventional CT units. As a consequence of scatter radiation, the soft tissue contrast is reduced and furthermore, Hounsfield units cannot be reliably measured [22]. Therefore, as an example, fluid-filled cystic lesions can only be differentiated from soft tissue masses if gas/fluid interfaces are present and based on other criteria such as their border definition, location, and extent. However, it remains to be elucidated if these limitations result in a clinically perceivable decrease in diagnostic yield when comparing CBCT and helical CT. Using cone beam CT, the spatial resolution should be slightly higher compared to conventional systems [22], but delineation of the lamina dura seemed difficult in some cases, especially in the presence of soft tissue attenuating content within the paranasal sinuses. The same has been reported using conventional CT [23]. Likely this is caused by insufficient spatial resolution.

The CBCT unit used has inherent and distinct advantages over conventional CT units for use in standing, sedated equids. One particular advantage is the mobility of the gantry, no necessity for a fixed installation or a high-voltage power supply. This allows movement of the machine to different rooms, and to adjust the gantry position in all three dimensions. Furthermore, the gantry can be opened around a surgery table, which makes the O-arm® an interesting imaging system also for intraoperative use. An advantage compared to robotic systems is that the gantry doesn’t move in relation to the patient, which increases the compliance of equids to the system.

We considered multiplanar reconstruction (MPR) in all different planes as very useful when assessing images. Interestingly, the image resolution is higher in the longitudinal axis compared to the transverse axis leading to images of better resolution in sagittal and dorsal planes. Furthermore, it seemed that motion affected the sagittal and dorsal reconstruction planes less compared with the transverse images, possibly due to the direction of the horses’ motion.

## Conclusions

We conclude that using a mobile CBCT scanner for head imaging is a valuable and feasible method in the standing sedated equid. In 97.3% of the horses, a radiological diagnosis could be established, or the presence of any structural changes could be excluded. Therefore, the diagnostic yield is comparable to that of conventional helical CT imaging in standing, sedated equids. Disadvantages of CBCT over conventional CT are the sensitivity to motion artefact, lower contrast resolution and the fixed field of view. Nonetheless, it is important to highlight the lower exposure to radiation as well as the lower costs for CBCT scanner acquisition.

## Methods

### Cadaver heads

Two cadaver heads from an adult horse and an adult donkey, both client-owned and euthanatized for reasons unrelated to this study, were collected.

The following ROI were defined based on location of swelling, abnormal findings in oral examination, presence and side of nasal discharge, and/or other additional information based on clinical examination and history: premaxilla/pars incisiva mandible, cheek teeth, sinus system, mandible, temporomandibular joint, middle and inner ear/proximal hyoid apparatus, and complete head, respectively. The minimum number of scans required to image the respective ROI was assessed.

### Clinically sound horses

**Fig. 5:**
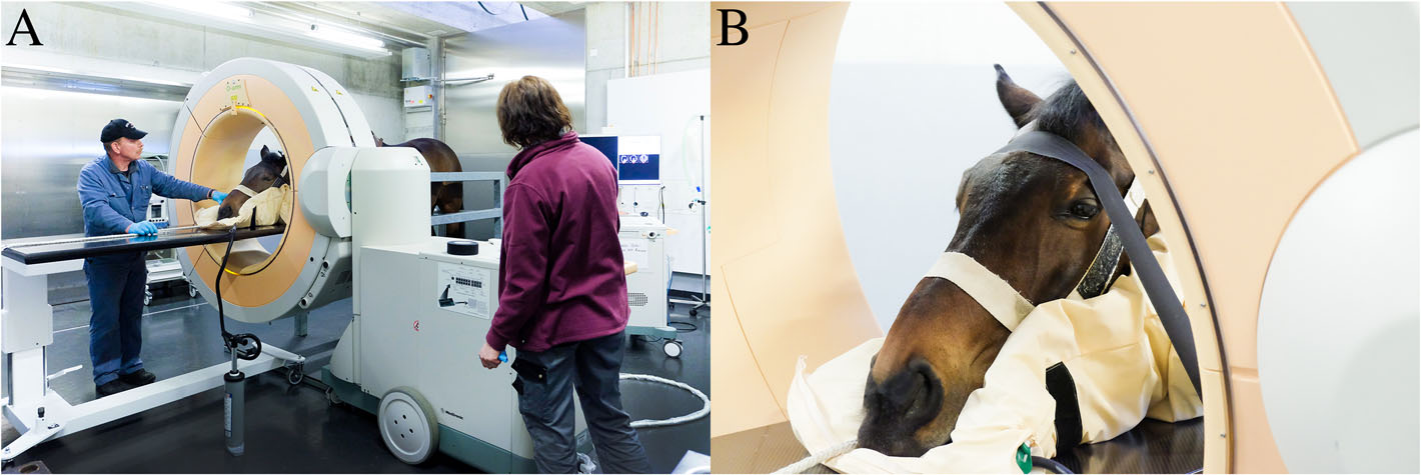
Positioning in stocks and head fixation of a horse for a CBCT scan in standing position. **a** Horse positioned in the custom-built stocks with the head resting in the vacuum cushion on a carbon table. Position, height, and tilt of the gantry are adjusted to the region of interest. **b** Head of the horse fixed with tape on the carbon table

The examinations in the teaching herd horses from the ISME equine clinic were performed under sedation using an initial bolus of detomidine (0.01 mg/kg intravenously; Equisedan, Dr. E. Graeub AG, Bern, Switzerland) [21]. Additional boluses of detomidine were administered as necessary. The use of the horses for this purpose was approved by the ethical committee (BE19/16). The ethical committee is a cantonal instance build up from experts evaluating all requests for animal experimentation in that region. Once the equid was adequately sedated, it was positioned in the custom-built stocks and secured with a leather strap crossing the back just caudal to the withers to avoid rearing up. Earplugs were used if tolerated by the subject. The head was positioned on a vacuum cushion (Philips AG Healthcare, Zurich, Switzerland), placed on a mobile carbon table adjustable in height (Raymed, Düdingen, Switzerland) (Fig. **5**a). Once the head was comfortably resting in the desired position, the vacuum was applied to shape the cushion in order to provide maximal stability to the resting head. Subsequently, the head was secured to the carbon table using adhesive tape (Tesaband®, Henry Schein, Lyssach, Switzerland) (Fig. **5**b). The effective length of the stocks could be adapted to the length of the horse with a movable hind bar. The front portion of the stocks was specifically designed so that the horse can lean against it.

Once the horse’s head was adequately and securely positioned, 2D fluoroscopic scout images were acquired in two different planes (laterolateral and dorsoventral or ventrodorsal, respectively). Based on the 2D scout images, position and orientation of the gantry were adjusted to the ROI.

All personnel were requested to leave the CT room during 2D and 3D image acquisition. Regardless of ROI, all scans of the head were performed using an exposure of 120 kV and 32 mAs.

### Clinical cases

The established examination protocol described for clinically sound horses was subsequently applied in all equids undergoing a standing CBCT examination of the head between February 2015 and November 2016. All horses were sedated with detomidine only (0.01 mg/kg intravenously as bolus; Equisedan, Dr. E. Graeub AG, Bern, Switzerland) [21]. If necessary additional boluses of detomidine were administred intravenously. Privately owned horses presented to the ISME equine clinic of Bern for the diagnosis and treatment of head disorders were included.

For each equid subjected to a standing CBCT examination of the head, signalment details, presenting complaints, and the indication or suspected underlying condition for imaging the ROI were recorded. Based on the presenting clinical complaints and clinical examination findings one or several ROIs were included in the CBCT examination.

For each equid, the total number of acquired 3D scans was recorded. One 3D scan corresponds to the cylindrical volume reconstructed by one rotation of the digital flat panel detector. Every scan was assessed by a certified radiology technician that decided whether or not to save the study for review or discard it because of obvious motion artefact, a technical problem during image acquisition or incomplete scan due to premature termination of 3D image acquisition (category IV) (Fig. **1**d). Every saved scan was then assessed for the presence of motion artefacts and assigned to one the following three categories: (I) no or minimal motion artefact, (II) mild motion artefact, (III) moderate motion artefact (Fig. **1**a-c).

All scans assigned to categories I through III were considered as scans of potentially diagnostic quality and therefore assessed by a board-certified radiologist using multiplanar reconstructions (MPR) in Impax EE R20 (Agfa HealthCare AG, Dübendorf, Switzerland). The number of radiological diagnosis and the number of cases without any structural changes were determined considering the clinical complaints/indications and based on their corresponding ROI imaged by CBCT.

## Data Availability

All image data and datasets used and/or analyzed during the current study are available from the corresponding author on reasonable request.

## Abbreviations

CBCT: cone beam computed tomography
CT: computed tomography
FDA: Food and Drug Administration
MPR: multiplanar reconstruction
ROI: region of interest
